# TGF-Beta Blockade Increases Renal Inflammation Caused by the C-Terminal Module of the CCN2

**DOI:** 10.1155/2015/506041

**Published:** 2015-05-05

**Authors:** Raquel Rodrigues-Díez, Sandra Rayego-Mateos, Macarena Orejudo, Luiz Stark Aroeira, Rafael Selgas, Alberto Ortiz, Jesús Egido, Marta Ruiz-Ortega

**Affiliations:** ^1^Cellular Biology in Renal Diseases Laboratory, School of Medicine, Universidad Autónoma Madrid, 28040 Madrid, Spain; ^2^Biomedical Research Center (CINBIO), Universidad De Vigo, 36282 Vigo, Spain; ^3^Dialysis Unit, IDIPAZ, 28046 Madrid, Spain; ^4^Dialysis Unit, IIS-Fundación Jiménez Díaz, Autonoma University, 28040 Madrid, Spain; ^5^Renal and Vascular Laboratory, IIS-Fundación Jiménez Díaz, Autonoma University, 28040 Madrid, Spain

## Abstract

The CCN family member 2 (CCN2, also known as connective tissue growth factor) may behave as a risk biomarker and a potential therapeutic target for renal disease. CCN2 participates in the regulation of inflammation and fibrosis. TGF-*β* is considered the main fibrogenic cytokine; however, in some pathological settings TGF-*β* also has anti-inflammatory properties. CCN2 has been proposed as a downstream profibrotic mediator of TGF-*β*, but data on TGF-*β* role in CCN2 actions are scarce. Our aim was to evaluate the effect of TGF-*β* blockade in CCN2-mediated experimental renal damage. Systemic administration of the C-terminal module of CCN2 to mice caused sustained renal inflammation. In these mice, TGF-*β* blockade, using an anti-TGF-*β* neutralizing antibody, significantly increased renal expression of the NGAL (a kidney injury biomarker), kidney infiltration by monocytes/macrophages, and upregulation of MCP-1 expression. The anti-inflammatory effect of TGF-*β* seems to be mediated by a dysregulation of the systemic Treg immune response, shown by decreased levels of circulating CD4^+^/Foxp3^+^Treg cells. Our experimental data support the idea that TGF-*β* exerts anti-inflammatory actions in the kidney and suggest that it is not an optimal therapeutic target.

## 1. Introduction

Chronic kidney disease (CKD) is a major health problem that has reached epidemic proportions and may lead to end-stage renal disease or early cardiovascular death [1]. Moreover, the increasing incidence of diabetes, hypertension, and obesity will result in future increases in the number of patients with CKD. Available therapy for CKD only delays, but does not prevent, disease progression. Besides, there are still no valid biomarkers that more accurately reflect the severity of the underlying renal histopathological changes and predict CKD progression or death [1]. Among the potential biomarkers and therapeutic targets, the CCN family member 2 (CCN2) has emerged as an interesting candidate [2]. CCN2 was initially described as the major platelet derived growth factor-related mitogen secreted by human vascular endothelial cells and named connective tissue growth factor (CTGF) [3]. This matricellular protein is a member of the CCN family of secreted, cysteine-rich regulatory proteins; therefore, the term CCN2 is used, as a proposal for uniform nomenclature [4]. CCN2 is a developmental gene, silenced in the adult kidney and reexpressed during kidney injury [2]. CCN2 levels in plasma or urine have been proposed to behave as risk biomarkers for CKD [5–7] and for cardiac dysfunction in patients exhibiting myocardial fibrosis and chronic heart failure [8]. Initial studies showed that CCN2 contributed to fibrosis [9], and it was proposed as an antifibrotic target [10, 11]. Experimental studies have shown that inhibition of endogenous CCN2 by antisense oligonucleotides slows disease progression in experimental diabetic nephropathy, unilateral ureteral obstruction, and nephrectomized TGF-*β*1 transgenic mice [3, 12–14], suggesting that selective CCN2 blockade could be used to treat renal disease.

CCN2 contains four functional modules than can be cleaved by proteases leading to several degradation products with biological activity [15]. Among these degradation fragments, the C-terminal cysteine knot module 4 of 11.2 kDa, named here CCN2(IV), has special relevance. CCN2 binds to the epidermal growth factor receptor (EGFR), through this C-terminal module [16]. Many studies have described that CCN2 and CCN2(IV) share many biological responses, including regulation of fibrosis [17], activation of the EGFR pathway, and downstream signalling, including MAPKs cascade [16]. Emerging experimental evidences support the novel concept of CCN2 as a proinflammatory cytokine [2, 18]. CCN2 is a chemotactic factor for immune cells [19], promotes cell adhesion and migration [2], and upregulates proinflammatory factors, including cytokines, chemokines, and adhesion molecules in resident cells [2, 18–21]. We have previously demonstrated that systemic administration of recombinant CCN2(IV) acutely induced Th1/Th2 cytokine production in the murine kidney after 24 hours, suggesting that CCN2 could induce inflammation* in vivo* [21]. Moreover, chronic CCN2(IV) administration caused a sustained kidney proinflammatory response, mainly characterized by activation of the Th17 immune response [19].

CCN2 as a mediator or coactivator of TGF-*β* mediated profibrotic responses [2, 9, 11, 22]. CCN2 overproduction has been proposed to play a major role in pathways that lead to fibrosis [2, 11]. Indeed, the notion that CCN2 is a downstream profibrotic mediator of TGF-*β* is the chief operating paradigm in the field, but there is no data on the effect of TGF-*β* blockade in CCN2 actions* in vivo*. In this paper we have investigated the effect of TGF-*β* blockade in experimental CCN2(IV)-induced renal damage, focusing on the regulation of inflammation and the modulation of Th17/Treg responses.

## 2. Materials and Methods

### 2.1. *In Vivo* Studies

Studies were performed in adult male C57BL/6 mice (9–12 weeks old, 20 g; obtained from Harlan Interfauna Ibérica) and maintained at the local animal facilities under special pathogen free conditions. All procedures on animals were performed according to the international and Instituto de Investigación Sanitaria Fundación Jiménez Díaz Animal Research Committee guidelines.

Mice received a single intraperitoneal injection (i.p.) of CCN2(IV) at the dose of 2.5 ng/g of body weight, dissolved in saline (*n* = 10 mice), as previously described [17, 18] and were studied 10 days later. The purity of CCN2(IV) (obtained from MBL/Peprotech, Bionova) was confirmed by MALDI-TOF (not shown). We have previously described that systemic CCN2(IV) administration caused a sustained inflammatory response in the kidney that peaked at 10 days [20]; therefore, this time point was chosen for the experiments. For TGF-*β* neutralization experiments, mice were injected with an anti-TGF-*β* pan-specific neutralizing antibody (100 *μ*g/mouse) or their corresponding IgG control (R&D, *n* = 10 mice per group), starting 24 h before CCN2(IV) injection and every 72 h thereafter until sacrifice at 10 days, following a previously described neutralization protocol [23].

Mice were sacrificed under anesthesia (Isoflurane, Abbott laboratories). The kidneys were perfused* in situ* with cold saline before removal. One kidney from each mouse was fixed in buffered formalin, embedded in paraffin, and used for immunohistochemistry. The other kidney was snap-frozen in liquid nitrogen for gene and protein studies.

### 2.2. Renal Histology and Immunohistochemistry

Paraffin-embedded sections were stained using standard histology procedures. Immunostaining was carried out in 3 *μ*m thick tissue sections that were deparaffinized and antigen retrieved using the PT Link system (Dako Diagnósticos) with Sodium Citrate Buffer (10 mM) adjusted to pH 6 or pH 9 depending on the immunohistochemical marker. Immunohistochemical staining was performed using the Dako Autostainer. The endogenous peroxidase was blocked and then sections were incubated for 30 min at room temperature with primary antibody: anti-CD3 and anti-CD4 (Dako) or anti-F4/80 (Serotec). After washing, slides were treated with the EnVision DuoFLEX Doublestain System using 3,3′-diaminobenzidine. For F4/80 staining, a rabbit anti-rat antibody was used as linker before EnVision treatment. Sections were counterstained with Carazzi's hematoxylin. The total number of positive stained cells was quantitated in 5 randomly chosen fields (200x) using Image-Pro Plus software. Data are expressed as positive stained area versus total analyzed area. Samples from each animal were examined in a blind manner. Negative controls were incubated with a nonspecific immunoglobulin of the same isotype as the primary antibody and without primary antibody.

### 2.3. Protein Studies

Kidney extracts were lysed in lysis buffer [50 mM Tris-HCl, pH 7.4, 150 mM NaCl, 2 mM EDTA, 2 mM EGTA, 0.2% Triton X-100, 0.3% NP40, 100 *μ*M phenylmethylsulfonyl fluoride, 1 mM dithiothreitol, 100 *μ*M Na_3_VO_4_, and 1 mM protease-inhibitor cocktail (Sigma)]. Protein concentration was determined by the BCA method (Pierce). Tissue protein extracts (30 *μ*g/lane) were separated on 8–12% polyacrylamide-SDS gels under reducing conditions. Samples were then transferred onto PVDF membranes (Bio-Rad), blocked in TBS with 0.05% Tween-20 and 5% nonfat dry milk, and then incubated overnight at 4°C with the primary antibodies and subsequently incubated with peroxidase-conjugated IgG (Amersham) and developed by ECL chemiluminescence (GE Healthcare). Autoradiographs were scanned using the GS-800 Calibrated Densitometer (Quantity One, Bio-Rad). Primary antibodies were affinity purified anti-mouse/human/rat Foxp3 (1 : 1000) (e-bioscience: 14-4774), NGAL (1 : 500) (Santa Cruz, sc-18698). The efficacy of protein loading and transfer to membranes was assessed by incubation with mouse anti-GAPDH antibody (1 : 5000) (Chemicon: MAB374). IL-17A levels were analyzed with an ELISA kit from eBioscience.

### 2.4. Gene Expression Studies

Total RNA was isolated from renal samples with Trizol (Invitrogen). cDNA was synthesized using the high capacity cDNA archive kit (Applied Biosystems) using 2 *μ*g of total RNA primed with random hexamer primers, following the manufacturer's instructions. Multiplex RT-PCR was performed using fluorogenic (FAM) TaqMan MGB probes and primers designed by Assay-on-Demand gene expression products (Applied Biosystems): MCP-1 Mm00441242_m1 and RANTES Mm01302428_m1. The mRNA copy numbers were calculated for each sample by the instrument software using Ct value. Results were expressed in copy numbers, calculated relative to control mice, after normalization against 18s (4210893E vic).

### 2.5. Flow Cytometry Analysis

In blood samples from different mice groups, circulating levels of T lymphocytes were evaluated. Cell surface staining was performed using FITC-labeled anti-CD3 or anti-CD8 and PE-labeled anti-CD4 (BD Pharmingen). After cell surface staining, Foxp3 was stained using Foxp3 staining kit (BD Pharmingen) according to manufacture instruction. Flow cytometry analysis was conducted on a FACSCalibur (BD Biosciences) with Cell Quest Pro software.

### 2.6. Statistical Analysis

Statistical analysis was done using the SPSS statistical software (version 11.0, Chicago, IL). After Kolmogorov-Smirnov test that determined the nonnormal sample distribution of the data, differences between groups were assessed by Mann-Whitney *U* test. The exact *P* value is shown in each graph bar.

## 3. Results

### 3.1. TGF-*β* Blockade Amplified CCN2(IV)-Induced Renal Inflammatory Response

**Figure 1:**
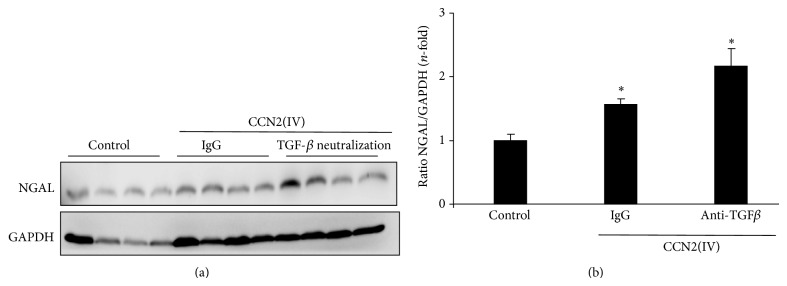
TGF-*β* blockade exacerbates CCN2(IV)-induced renal damage. C57BL/6 mice received a single ip injection of 2.5 ng/g body weight recombinant CCN2(IV) or vehicle (saline) and were sacrificed after 10 days. For TGF-*β* neutralization experiments, mice were treated with anti-TGF-*β* antibody or an isotype IgG control (*n* = 10 mice per group) starting 24 hours before CCN2(IV) injection and every 72 h until sacrifice at day 10 after CCN2(IV). Renal damage was assessed by evaluation of renal levels of biomarker NGAL by western blot. (a) shows a representative western blot and data expressed as mean ± SEM (*n* = 10 animals per group) of fold-change as compared to controls. ^∗^ *P* < 0.05 versus control.

To evaluate the effect of TGF-*β* blockade on CCN2(IV)-induced renal damage, active TGF-*β* was blocked using a pan-specific neutralizing anti-TGF-*β* antibody or its corresponding isotype IgG, and renal damage was evaluated by assessment of the biomarker of renal injury, neutrophil gelatinase-associated lipocalin (NGAL) [24]. In CCN2(IV)-injected mice, kidney NGAL protein expression levels were elevated showing a significant upregulation in the CCN2(IV)-injected TGF-*β*-treated mice (Figure 1). These data suggest that TGF-*β* blockade increased CCN2(IV)-mediated renal damage.

**Figure 2:**
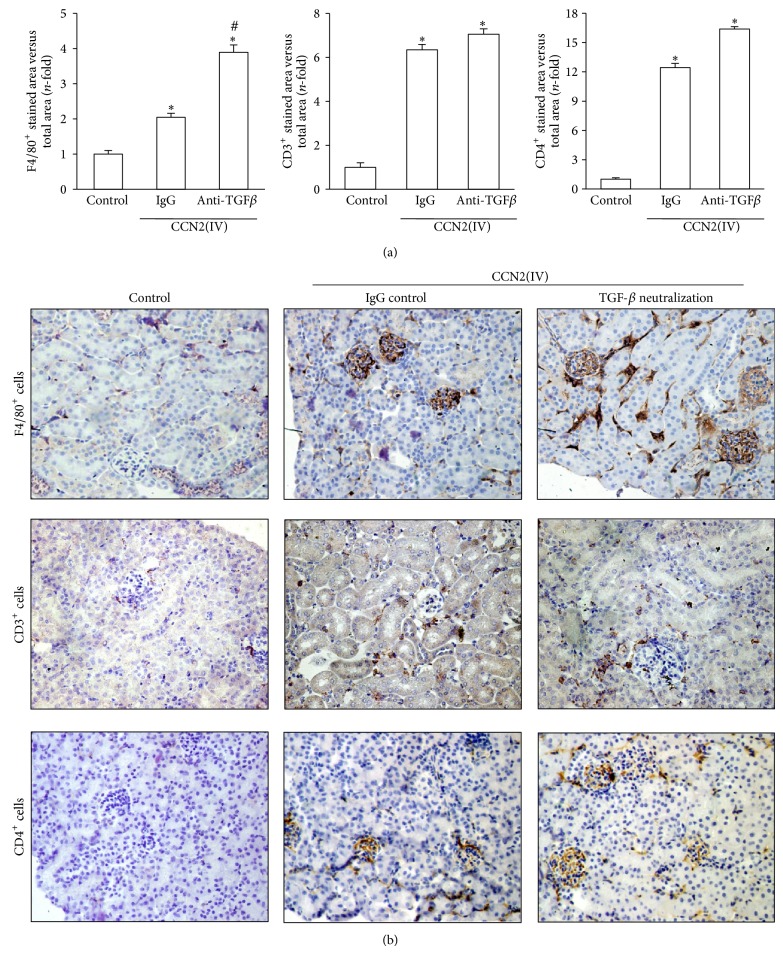
TGF-*β* neutralization increases CCN2(IV)-induced renal inflammatory response. The inflammatory cell infiltration was characterized in paraffin-embedded renal sections by immunohistochemistry with anti-F4/80 (specific for monocyte/macrophage), anti-CD3 (T lymphocyte marker), and anti-CD4 (effector lymphocyte T marker) antibodies. (a) shows the immunohistochemistry quantification expressed as mean ± SEM (*n* = 10 animals per group) of fold-change as compared to controls. Mean ± SEM. ^∗^ *P* < 0.05 versus control. ^#^ *P* < 0.05 versus CCN2(IV)-IgG. (b) shows a representative animal from each group (200x magnification). Arrows indicate infiltrating cells in detail (400x magnification).

**Figure 3:**
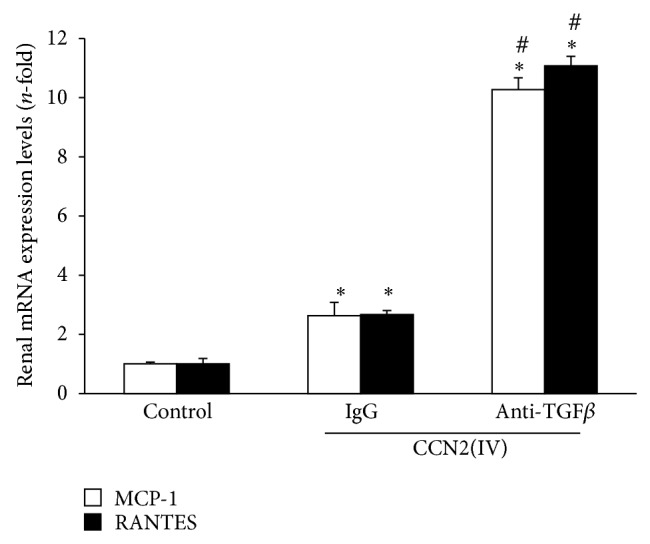
TGF-*β* blockade upregulates CCN2(IV)-induced renal chemokine expression. Kidney gene expression of MCP-1 and RANTES was evaluated by real time PCR. Data are expressed as *n*-fold increase over control as mean ± SEM of 10 animals per group. ^∗^ *P* < 0.05 versus control. ^#^ *P* < 0.05 versus CCN2(IV)-IgG.

The main pathological feature of CCN2(IV) administration to mice was kidney infiltration by leukocytes and upregulation of proinflammatory mediators, while renal fibrosis was not observed [20]. Therefore, we evaluated the effect of TGF-*β* blockade on CCN2(IV)-induced renal inflammation. Treatment with a TGF-*β* neutralizing antibody significantly increased the number of infiltrating inflammatory cells, mainly F4/80^+^ monocytes-macrophages, in the kidney of CCN2(IV)-injected mice, compared to IgG-treated CCN2(IV)-injected mice (Figures 2(a) and 2(b)). Moreover, renal gene expression of several proinflammatory mediators was elevated in response to TGF-*β* neutralization (Figure 3). The recruitment of immune cells into damaged tissue is mainly regulated by chemokines. Among them, MCP-1 is the main chemokine involved in monocytes-macrophages recruitment [25]. In CCN2(IV)-injected mice, renal MCP-1 and RANTES gene expression was significantly increased in response to TGF-*β* blockade (Figure 3). Moreover, MCP-1 protein levels were also upregulated in this group (around 2-fold versus IgG-treated CCN2(IV)-injected mice, data not shown). These data suggest an activation of the local inflammatory response when TGF-*β* is blocked and confirm the role of TGF-*β* as an anti-inflammatory cytokine.

### 3.2. TGF-*β* Blockade Modulates Systemic Treg, but Not Th17, Immune Response in CCN2(IV)-Injected Mice

**Figure 4:**
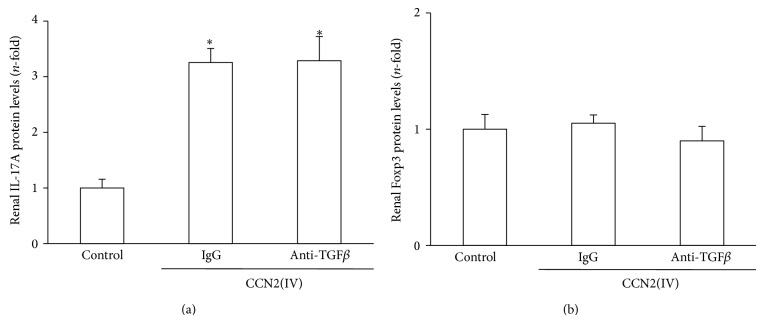
Effect of TGF-*β* blockade on renal Th17/Treg responses modulated by CCN2(IV). The main markers for Th17 or Treg were evaluated in renal total protein extracts. (a) Renal levels of IL-17A were evaluated by ELISA. (b) Foxp3 renal levels were analysed by western blot. Data is shown as ratio of renal Foxp3/GAPDH protein. Mean ± SEM of 10 mice per group and representative western blot experiment. ^∗^ *P* < 0.05 versus control.

TGF-*β* behaves as an anti-inflammatory factor in some conditions and it has been involved in the differentiation of Treg cells, through activation of the transcription factor X-linked forkhead/winged helix (Foxp3) [26, 27]. We have previously reported that Th17, but not Treg, immune response participates in CCN2(IV)-mediated renal inflammation [20]. Our next aim was to evaluate whether the effect of TGF-*β* blockade on the renal inflammatory response could be mediated by a Th17/Treg response imbalance. In CCN2(IV)-injected mice, TGF-*β* blockade did not modify IL17A (Figure 4(b)) or Foxp3 (Figure 4(a)) renal levels.

**Figure 5:**
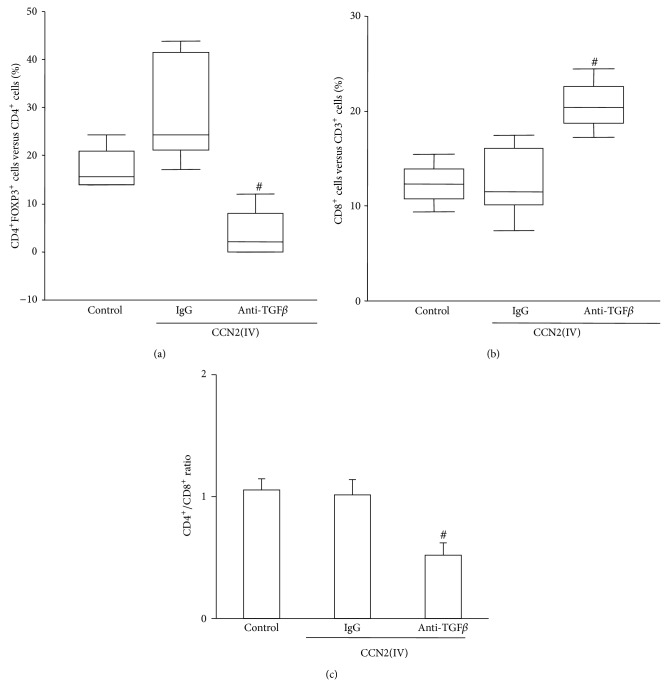
TGF-*β* blockade modulates circulating immune cells. Lymphocyte populations were analyzed in blood samples by flow cytometry. (a) represents the percentage of CD4^+^ FOXP3^+^ cells among CD4^+^ T cells. (b) shows the percentage of cytotoxic (CD8^+^) T lymphocytes among total CD3^+^ T lymphocytes. The CD4^+^/CD8^+^ ratio is shown in (c) (mean ± SEM of 5 mice per group). ^#^ *P* < 0.05 versus CCN2(IV)-IgG.

We analysed the number of circulating blood cells by flow cytometry. Although circulating CD4^+^/Foxp3^+^ Treg cells were not modified in CCN2(IV)-injected mice, TGF-*β* blockade dramatically decreased circulating CD4^+^/Foxp3^+^ Treg cells (Figure 5(a)). In addition, TGF-*β* blockade increased the number of T cytotoxic lymphocytes (CD8^+^) and decreased the CD4^+^/CD8^+^ ratio (Figures 5(b) and 5(c)). These data suggest that TGF-*β* blockade could lead to a systemic inflammatory response that increases the kidney susceptibility to inflammation.

## 4. Discussion

In this report we described that TGF-*β* blockade increased the kidney inflammatory response to CCN2(IV) administration. This increased inflammatory response was mainly characterized by the local kidney upregulation of MCP-1 and proinflammatory chemokines and the infiltration of monocytes/macrophages as well as by the dysregulation of the systemic Treg immune response.

CCN2 regulates numerous cellular processes including cell differentiation, adhesion, proliferation, and, as we described here, inflammation. CCN2 is a multimodular protein of four functional modules: the N-terminal insulin-like growth factor-binding domain (IGFB), the cysteine-rich domain (also called von Willebrand type c domain), a thrombospondin type 1 repeat domain, and the C-terminal heparin-binding domain [2, 28]. Functional domains within the CCN2 modules interact with different growth factors, receptors, and matrix components and mediate specific CCN2 responses [2, 28]. These modules may be cleaved by proteases to yield several biologically active degradation products [15]. In chondrocytes, the N-terminal CCN2 module, but not the C-terminal module, had a direct interaction with the proteoglycan aggrecan and stimulated its production [29]. In Xenopus cells, the N-terminal module, through the cysteine-rich domain, can directly bind to TGF-*β* in the extracellular space, potentiating TGF-*β* receptor binding and Smad signalling [30]. In cultured mesangial cells, CCN2, via its N-terminal module, antagonized TGF-*β*1 binding to TGF-*β* type III receptor (endoglin) and inhibited Smad pathway activation [31]. The thrombospondin type 1 repeat domain can bind to extracellular matrix and vascular endothelial growth factor [2]. Some authors have hypothesized that CCN2 is a downstream mediator of TGF-*β* profibrotic actions [10, 11]. Individual CCN2 domains interact with TGF-*β* in a different manner: the C-terminal domain mediated TGF-*β*-induced fibroblast proliferation, whereas the N-terminal domain mediated myofibroblast differentiation and collagen synthesis [32]. The C-terminal CCN2 module binds integrins and regulates signalling in fibrosis and inflammation [2, 10]. We have recently demonstrated that the C-terminal module of CCN2 binding to EGFR regulates renal and vascular inflammation [16, 33]. In tubular epithelial cells, *α*V*β*3 integrin directly binds to the C-terminal CCN2 module but is not necessary for the binding of CCN2 to EGFR and the subsequent complex formation [16]. All these data show the complexity of CCN2 actions and the importance of the evaluation of CCN2 modules functional activities.

Although many* in vitro* studies have demonstrated that TGF-*β*1 induces CCN2 synthesis [11, 12], there are scarce studies evaluating whether CCN2 could regulate TGF-*β* expression or actions. In addition, TGF-*β* synthesis is a complex process with multiple steps of regulation [12]. Our group has previously described that systemic Angiotensin II infusion upregulates tissue CCN2 as early as at 3 days, while elevated levels of active TGF-*β*1 were observed later on, at 7 days. Moreover, CCN2 induction was associated with inflammation, while TGF-*β*1 overproduction correlated with fibrosis, as assessed by fibronectin and collagen deposition [34–36]. Accordingly, CCN2(IV) administration in mice caused a sustained renal inflammation, but there was no increase in TGF-*β* synthesis or matrix deposition [20]. These data suggest that CCN2* in vivo* could be involved in the induction of a proinflammatory or a profibrotic response depending on the presence/absence of TGF-*β*1.

TGF-*β* is a pleiotropic cytokine that has been involved in many human diseases, including proliferative disorders, fibrotic diseases, and immune-mediated pathologies [11, 37]. TGF-*β*1 is a key factor in fibrosis, including the kidney fibrosis [38–43]. In cultured renal cells, TGF-*β*1 stimulates extracellular matrix production, inhibits matrix degradation by the regulation of matrix metalloproteinases, and is a key factor in the induction of tubuloepithelial to mesenchymal transition [42, 43]. Increased levels of active circulating TGF-*β*1 in mice caused renal fibrosis [38]. TGF-*β* blockade ameliorated experimental fibrosis in models of vascular restenosis, spontaneously hypertensive rats [11], and peritoneal damage [39]. Both in CKD patients and in experimental renal fibrosis, elevated renal levels of TGF-*β*1 have been associated with fibrosis, characterized by excessive matrix accumulation in the glomerulus and in the interstitium [40, 44–50]. TGF-*β*1 blockade by different approaches, including neutralizing antibodies, siRNAs, or blockers such as decorin, inhibited fibrosis both* in vitro* and in experimental renal disease [40, 50–53]. Interestingly, in some of these models, such as puromycin-induced nephrosis or diabetic nephropathy, TGF-*β* blockade worsened both proteinuria and albuminuria [40, 50–53]. Furthermore, in unilateral urethral obstruction, conditional deletion of TGF-*β*1 ameliorated tubulointerstitial fibrosis but increased inflammation [54]. In this paper, we have observed that TGF-*β*1 neutralization increased experimental renal inflammation induced by CCN2(IV), and this was mainly characterized by a significant increase in local kidney proinflammatory chemokines and macrophage infiltration.

Beside its role as a profibrotic factor, TGF-*β* has anti-inflammatory functions. Indeed, mice deficient in TGF-*β*1 develop a lethal multiorgan inflammatory disease and died at 3-4 weeks of age [55], and conditional deletion of TGF-*β*1 or its type II receptor in T cells induced autoimmune disease [56, 57]. Moreover, increased TGF-*β* plasma levels as a consequence of either exogenous administration or overexpression protect from experimental inflammatory diseases, including arthritis, autoimmune encephalomyelitis, nonobese diabetic mice, and systemic lupus erythematous [58–60]. Moreover, mice overexpressing latent TGF-*β* were protected against both renal inflammation and fibrosis in obstructive kidney disease models [41, 61]. In experimental vascular damage, TGF-*β* blockade caused local inflammation associated with no reduction in stent-induced neointima formation [62] or acceleration of atherosclerotic plaque formation [63–65].

The anti-inflammatory actions of TGF-*β*1 have been attributed to its role in the activation of Treg response [66, 67]. Treg cells can suppress immune responses to autoantigens, alloantigens, and infectious agents [68]. In several experimental models, Treg cell administration was beneficial. In experimental Angiotensin II infusion, intravenous administration of Treg cells inhibited immune cell infiltration and decreased proinflammatory mediators in both renal and vascular tissues [69]. Treg cells injection inhibited experimental renal damage, including anti-GBM glomerulonephritis and adriamycin-induced nephropathy [70, 71]. Patients with lupus nephritis presented elevated Th17 immune response and exhibited low systemic levels of TGF-*β*1 and Treg cells, compared to healthy subjects [72, 73]. We have observed that neutralizing antibodies against TGF-*β* downregulated circulating CD4^+^/Foxp3^+^ Treg cells in experimental CCN2(IV)-mediated renal damage, suggesting that TGF-*β* blockade significantly impaired the protective effect of Treg cells.

We have previously described that systemic long-term CCN2(IV) administration induced a local sustained Th17 immune response, characterized by increased kidney IL-6 production, ROR*γ*t levels, and STAT3 activation, with no changes in renal levels of Th1/Th2 cytokines or Treg-related factors (TGF-*β* and foxp3), suggesting that Th1/Th2/Treg responses were not modulated by CCN2(IV), at least in the murine kidney [17]. Now, we have observed that TGF-*β* blockade did not modify Th17 immune response, as shown by unaltered renal IL-17A levels. Moreover, renal Foxp3 levels, which regulate Treg differentiation, were not changed. Our data indicates that TGF-*β* blockade did not modify renal levels of Th17/Treg differentiation factors, suggesting that the anti-inflammatory effects could be mediated by the regulation of systemic Treg levels.

## 5. Conclusions

Our experimental data support the idea that TGF-*β* exerts anti-inflammatory actions in the kidney and suggest that TGF-*β* blockade may not be an adequate therapeutic strategy for kidney disease.
